# Effects of PPARα inhibition in head and neck paraganglioma cells

**DOI:** 10.1371/journal.pone.0178995

**Published:** 2017-06-08

**Authors:** Rosalba Florio, Laura De Lellis, Viviana di Giacomo, Maria Carmela Di Marcantonio, Loredana Cristiano, Mariangela Basile, Fabio Verginelli, Delfina Verzilli, Alessandra Ammazzalorso, Sampath Chandra Prasad, Amelia Cataldi, Mario Sanna, Annamaria Cimini, Renato Mariani-Costantini, Gabriella Mincione, Alessandro Cama

**Affiliations:** 1 Department of Pharmacy, "G. d'Annunzio" University of Chieti-Pescara, Chieti, Italy; 2 Unit of General Pathology, CeSI-MeT, “G. d’Annunzio” University, Chieti, Italy; 3 Department of Medical, Oral and Biotechnological Sciences, "G. d'Annunzio" University of Chieti-Pescara, Chieti, Italy; 4 Department of Life, Health and Environmental Sciences, University of L’Aquila, L’Aquila, Italy; 5 Department of Otology and Skull Base Surgery, Gruppo Otologico, Piacenza, Italy; 6 Sbarro Institute for Cancer Research and Molecular Medicine, Temple University, Philadelphia, United States of America; 7 Gran Sasso National Laboratory (LNGS), National Institute for Nuclear Physics (INFN), Assergi, Italy; University of South Alabama Mitchell Cancer Institute, UNITED STATES

## Abstract

Head and neck paragangliomas (HNPGLs) are rare tumors that may cause important morbidity, because of their tendency to infiltrate the skull base. At present, surgery is the only therapeutic option, but radical removal may be difficult or impossible. Thus, effective targets and molecules for HNPGL treatment need to be identified. However, the lack of cellular models for this rare tumor hampers this task. PPARα receptor activation was reported in several tumors and this receptor appears to be a promising therapeutic target in different malignancies. Considering that the role of PPARα in HNPGLs was never studied before, we analyzed the potential of modulating PPARα in a unique model of HNPGL cells. We observed an intense immunoreactivity for PPARα in HNPGL tumors, suggesting that this receptor has an important role in HNPGL. A pronounced nuclear expression of PPARα was also confirmed in HNPGL-derived cells. The specific PPARα agonist WY14643 had no effect on HNPGL cell viability, whereas the specific PPARα antagonist GW6471 reduced HNPGL cell viability and growth by inducing cell cycle arrest and caspase-dependent apoptosis. GW6471 treatment was associated with a marked decrease of CDK4, cyclin D3 and cyclin B1 protein expression, along with an increased expression of p21 in HNPGL cells. Moreover, GW6471 drastically impaired clonogenic activity of HNPGL cells, with a less marked effect on cell migration. Notably, the effects of GW6471 on HNPGL cells were associated with the inhibition of the PI3K/GSK3β/β-catenin signaling pathway. In conclusion, the PPARα antagonist GW6471 reduces HNPGL cell viability, interfering with cell cycle and inducing apoptosis. The mechanisms affecting HNPGL cell viability involve repression of the PI3K/GSK3β/β-catenin pathway. Therefore, PPARα could represent a novel therapeutic target for HNPGL.

## Introduction

Head and neck paragangliomas (HNPGLs) are rare neuroendocrine tumors, originating from paraganglia associated with automic branches of the lower cranial nerves [1]. They account for about 0.6% of all head and neck tumors and usually present between the 4^th^ and 6^th^ decades of life. HNPGLs mostly arise from paraganglia at the carotid bifurcation, in or around the jugular bulb, in the cervical tract of the vagus, or within the temporal bone. HNPGLs are generally slow-growing, but they infiltrate vascular structures and anatomically complex regions of the skull base. A high percentage of HNPGLs (over 30%) arises on the basis of genetic predisposition [1]. Germline mutations in the succinate-ubiquinone oxidoreductase (succinate dehydrogenase, SDH) subunits are the most frequently involved in HNPGL predisposition. The corresponding genes encode subunits (*SDHA*, *SDHB*, *SDHC*, *SDHD)* or cofactors (*SDHAF2*) of Complex II succinate dehydrogenase. This complex of the mitochondrial respiratory chain is responsible for the enzymatic oxidation of succinate to fumarate in the tricarboxylic acid cycle and for electron transport to coenzyme Q [2, 3]. At present, surgery is the only therapeutic option, but in some case radical removal may be difficult or impossible, depending on location and stage of progression [4]. When surgical eradication is not possible, radio- and chemotherapy are used, but only partial responses are observed even with targeted radiotherapy [5]. Thus, novel molecular targets and/or molecules that could be exploited in HNPGL therapy are highly needed. However, little is known about the effects of molecules with therapeutic potential in HNPGLs, also due to the lack of commercially available cell lines for this rare tumor. In a previous study we developed a primary human HNPGL cell culture and showed that lentiviral expression of tumor suppressor miRNAs downregulated in those cells was associated with apoptosis activation and cytotoxicity [6].

Peroxisome Proliferator-Activated Receptors (PPARs) are ligand-activated transcription factors involved in the regulation of glucose and lipid homeostasis, inflammation, proliferation, differentiation and cell death [7, 8]. Following ligand binding, PPARs heterodimerize with retinoid X receptor (RXR) triggering the transcription of a variety of target genes. The three PPAR subtypes, PPARα, PPARγ and PPARβ/δ, are often activated in tumors, where these receptors appear to modulate cell proliferation, differentiation and survival, supporting an important role of PPARs in cancer biology [8]. Among PPARs, PPARα receptors appear to the have an important, but pleiotropic role in malignancy. Whether they function as tumor suppressors or promoters in cancers, it is context-dependent. Indeed, such functions appear to be related to cancer type and/or specific microenvironment of the tumor. In this regard, PPARα receptor activation was reported in several tumors, including hepatocellular carcinoma [9], breast cancer [10], glioblastoma [11, 12], chronic lymphocytic leukemia (CLL) [13] and kidney cancer [14]. In particular, we have previously shown that PPARα is strongly upregulated in high-grade glioma [15]. Moreover, PPARα-deficient mice were refractory to liver carcinogenic effects of the PPARα agonist WY14643 [16]. In addition, growth and progression of lung carcinoma and melanoma tumors engrafted in wildtype mice were completely suppressed when these tumors were implanted in PPARα-deficient mice [17]. These reports indicated PPARα as a promising target for the treatment of cancer. To this end, a few PPARα antagonists are available and some have been shown to exert beneficial effects in cancer [18–20]. However, to date, no selective PPARα antagonists have been tested in human trials.

The role of PPARα in HNPGLs is not known. In the present study we evaluated the expression of PPARα and the effects of stimulating or inhibiting this receptor with a specific agonist (WY14643) or antagonist (GW6471), respectively, in a unique model of HNPGL cells. Our results show that PPARα is highly expressed in HNPGL cells and that the antagonist GW6471 reduces viability of HNPGL cells through mechanisms involving cell cycle arrest and apoptosis.

## Materials and methods

### Reagents and antibodies

The PPARα antagonist GW6471 was obtained from Tocris Bioscience (Bristol, UK). 3-(4,5-Dimethyl-2-thiazolyl)-2,5-diphenyl-2H-tetrazolium bromide (MTT), l-glutamine, 4’,6 diamino- 2-phenylindole dilactate (DAPI), crystal violet, the PPARα agonist WY14643, the RNAse and the propidium iodide (PI) solution were obtained from Sigma (St. Louis, MO, USA). Mouse monoclonal anti-cyclin D3 antibody, rabbit monoclonal anti-CDK4 antibody, rabbit monoclonal anti-p21Waf1/Cip1 antibody, anti-rabbit IgG/HRP-linked and anti-mouse IgG/HRP-linked were purchased from Cell Signaling Technology, Inc. (Beverly, MA, USA). Rabbit monoclonal antibodies anti-PI3K and anti-GSK3β were obtained from Abcam (Cambridge, UK). Rabbit polyclonal anti-pser9GSK3β was obtained from Enogene Biotech. (Aachen, Germany). Mouse monoclonal antibody anti-cyclin B1, rabbit polyclonal anti-β-Catenin and mouse monoclonal anti-glyceraldehyde-3-phosphate dehydrogenase (GAPDH) were obtained from Santa Cruz Biotechnology, Inc. (Dallas, TX, USA). Monoclonal anti-β-actin antibody was obtained from Sigma (St. Louis, MO, USA). Rabbit anti-PPARα and rabbit anti-PPARγ were obtained from Thermo Scientific (Rockford, IL, USA). Rabbit anti-PPARβ/δ was obtained from Affinity Bioreagents Inc. (Golden, CO, USA). Anti-rabbit IgG Alexa Fluor 488 conjugated secondary antibodies were purchased from Molecular Probes (Life Technology, Carlsbad, CA, USA.

### Cells and treatments

Primary HNPGL cell cultures were established as previously described [6] from two prospectively sampled tympano-jugular HNPGL patients (PTJ64 and PTJ86) carrying the *SDHC* c.43C>T (p. Arg15*) and *SDHD* c. 27delC (p. Val10Phefs*5) mutations, respectively. For mutational analysis the coding regions and exon–intron boundaries of *SDHB*, *SDHC* and *SDHD* genes were amplified by PCR as previously described [6, 21, 22]. PCR products were subjected to 2% agarose gel electrophoresis with ethidium bromide staining and subsequently sequenced using a genetic analyzer (ABI PRISM 310; Applied Biosystems, Milan, Italy). Biospecimens from which primary cultures derived were collected after written informed consent from patients operated at the Gruppo Otologico, Piacenza, Italy. Study protocols and consent forms were approved by the Bioethical Committee of G. d’Annunzio University (protocol #841/10COET). The cultures were immortalized by retroviral-mediated transduction of full-length hTERT and simian virus 40 (SV40) large-tumor (LT) antigen (Addgene, https://www.addgene.org/). The hTERT virus was constructed by co-transfecting vectors bringing cDNAs for Gag-polymerase, virus envelope proteins and full-length hTERT (pBabe-hygro-hTERT) into HEK293 cells. An analogue procedure was followed to construct the SV40LT virus using the pBabe-puro SV40LT vector. Mid-confluence HNPGL primary cultures at passage 4 were exposed for 3–6 hours to filtered (0.4 μm) supernatants from the retroviral packaging cell line containing the virus construct for SV40LT, in the presence of polybrene (5 μg/ml). Infected HNPGL cells were incubated with puromycin to select SV40LT transduced cells. These cells were grown for two passages before the second infection with supernatants from the retroviral packaging cell line containing the virus construct hTERT and cells transduced with hTERT were selected by hygromycin B. Immortalized HNPGL cells (PTJ64i and PTJ86i, respectively) cultured in DMEM-F12 (Gibco), supplemented with 10% FBS, at 37°C, 5% CO_2,_ were employed in subsequent experiments.

Stock solutions of GW6471 (70 mM) and WY14643 (35 mM) were prepared in DMSO. The final concentration of DMSO in experiments was 0.16% and showed no HNPGL cell toxicity.

### Immunohistochemistry

Two different HNPGL cases were analyzed for immunohistochemistry (IHC). Sections were deparaffinized by using xylene and graded ethanol and rehydrated. Slides were then immersed in 10mM sodium citrate buffer, pH 6.1, and processed for the antigen-retrieval procedure, using a microwave oven operated at 720W, for 10 min. After cooling, slides were transferred to phosphate buffer saline (PBS) containing 5% bovine serum albumin (BSA, blocking solution), for 1h at room temperature and then incubated, overnight at 4°C, with rabbit polyclonal anti-PPARα antibody (1:200, Thermo Scientific Inc., USA) diluted in blocking solution. In control sections, the primary antibody was omitted. Slides were then incubated for 1h at RT with biotinylated goat anti-rabbit IgG (1:200, Vector Laboratories, Burlingame, CA, USA), diluted in blocking solution. Immuno-complexes were revealed by means of an avidin biotin system (Vectastain Elite ABC kit, Vector Laboratories), using 3,3’-diamino-benzidine (DAB Substrate kit for Peroxidase, Vector Laboratories), as the chromogen. Slides were finally dehydrated and mounted with Eukitt (Kindler GmbH & Co., Freiburg, Germany).

### Immunofluorescence

Cells plated on poly-L-lysine coated coverslip were washed twice with PBS, fixed for 10 minutes at room temperature in 4% paraformaldehyde in PBS and permeabilized in PBS containing 0.1% Triton X-100 for 10 min at room temperature. Nonspecific binding sites were blocked for 30 min with 3% BSA in PBS (incubation buffer). According to the experiment, cells were then incubated with anti-PPARα (1:200), or anti- PPARβ/δ (1:200) or anti-PPARγ (1:200) in incubation buffer overnight at 4°C. After extensive washings with PBS the cells were incubated with AlexaFluor 488 secondary antibodies 30 min at room temperature. Cells were then washed and mounted with Vectashield mounting medium from Vector Laboratories containing DAPI. Cells were photographed at fluorescence microscope AXIOPHOT (Zeiss microscope, Jena, Germany).

### Cell viability and counting

Cell viability was tested by MTT assay as previously described [23]. The dose and the schedule of GW6471 or WY14643 treatments were selected based on dose–response curves constructed in preliminary experiments. Briefly, cells were seeded in 96-well plates (4x10^3^ cells/well) and incubated with the indicated treatments (5 replica wells per each condition) for 72 hours. Then, cells were incubated with the MTT solution for at least 3 hours, until a purple precipitate was visible. Then, the MTT solution was removed and crystalline precipitate in each well was dissolved in DMSO. Absorbance of each well at 570 nm was quantified using a microplate reader (SoftMaxPro, Molecular Devices, CA, USA). For cell counting, HNPGL cell cultures were treated with GW6471 (24 μM) or vehicle (DMSO) in triplicate. After seeding (9.5x10^5^ cells/75 cm^2^ flask), cells were incubated with GW6471 or vehicle and viable cells were counted at 24, 48 and 72 hours using the trypan blue exclusion test [24].

### Cell cycle analysis

Approximately 0.5 x 10^6^ cells per experimental condition were harvested, fixed in 70% cold ethanol and kept at 4°C overnight. Cells were then resuspended in 20 μg/ml PI and 100 μg/ml RNAse, final concentrations. Cell cycle profiles (10^5^cells) were analyzed by a FC500 flow cytometer with the FL3 detector in a linear mode using the CXP software (Beckmann Coulter, FL, USA). Data were analyzed with ModFit software (Verity Software House, ME, USA).

### Apoptosis assay

To assess apoptosis a commercial Annexin-V-FITC/PI Kit (Bender Med System, Vienna, Austria) was used according to the manufacturer’s instructions. Briefly, the cells were gently resuspended in binding buffer and incubated for 10 min at room temperature in the dark with Annexin-V-FITC. Samples were then washed and analyses were performed with an FC500 Coulter flow cytometer with the FL1 detector in a log mode using the CXP analysis software (both from Beckmann Coulter). For each sample, at least 10^5^ events were collected. Viable cells are Annexin-V^neg^ while apoptotic cells are Annexin-V^pos^.

### Caspase 3–7 activity assay

Apoptotic effects of 24 μM GW6471 treatments were measured using the Caspase-Glo 3/7 assay (Promega, Madison, WI, USA), according to manufacturer’s instruction. Briefly, cells were seeded in triplicate in 96-well plates (5x10^3^ cells/well). After an overnight incubation, cells were incubated with the drug for 24 and 72 hours. At the time of apoptosis measurement, plates were allowed to reach room temperature for 30 minutes, then cells were incubated with the Caspase-Glo 3/7 assay reagent (100 μl for 100 μl of medium per well). Plates were covered, placed on a shaker for approximately 30 seconds and incubated at room temperature for 30 minutes. Luminescence at 100 nm was analyzed using the VERITAS microplate luminometer (Turner BioSystems) and caspase activity was measured by averaging six determinations.

### Western blotting analysis

Immunoblotting was performed as previously described [23]. Briefly, after treatments the cells were washed with PBS and cell lysates were cleared by centrifugation at 12.000g for 20 minutes. Protein concentrations were determined by the BCA Protein Assay (Thermo Scientific, Rockford, IL, USA), then protein lysates were subjected to electrophoresis followed by immunoblotting. For CDK4, cyclin B1, Cyclin D3 and p21 immunoblotting 30 μg of protein lysates were used, whereas for PI3K, GSK3β, pser9GSK3β and β-catenin immunoblotting 50 μg/lane of protein lysates were used. The membranes were blocked in 5% nonfat dry milk for one hour at room temperature and incubated overnight at 4°C with the appropriate primary antibodies. Then the membranes were incubated with either anti-rabbit or anti-mouse (1:1000) HRP-conjugated secondary antibodies. The blots were revealed by chemiluminescence using the SuperSignal West Pico Chemiluminescence Substrate (Thermo Scientific, Rockford, IL, USA) according to the manufacturer's instructions. β-actin or GAPDH were used as loading controls.

### Clonogenic assay

Clonogenic assay was performed essentially as described by Franken et al. [25]. Based on preliminary experiments with four different seeding concentrations (100, 200, 300 and 400 cells per well), we chose a concentration of 200 cells per well in a 6-well plate. Following cell attachment we treated cells for 3 days with 24 μM GW6471 and then, after medium refreshment, we left the plate in the incubator at 37°C, 5% CO_2_, until cells in the control vehicle formed colonies consisting of at least 50 cells (12–15 days). Colonies were fixed with 6% glutaraldehyde and stained with 0.5% crystal violet, then rinsed with tap water, dried and counted.

### *In vitro* wound-healing assay

Tumor cells in medium containing 10% FBS were seeded in 12-well plates (2x10^5^ cells/well). After the cells grew to confluence, wounds were made by sterile pipette tips. Plates were washed twice with PBS in order to remove the detached cells and incubated with medium containing 24 μM GW6471 or with vehicle. Pictures of the cultures were taken at 0 h (immediately after scratching) and at the indicated time intervals, until the wound closure was completed by cells treated with vehicle. The number of cells that migrated from the wound edge into the uncovered area was quantified in three fields per well. Data were expressed as ratio of migrated cells after GW6471 treatment versus vehicle.

### Statistical analysis

Comparisons of mean values were performed by the independent samples t-test using the Dunnett’s test for multiple comparisons where appropriate. A *p*-value of 0.05 was considered statistically significant. IC_50_ values were calculated using the CompuSyn software [26].

## Results

### PPARα is highly expressed in HNPGL tissues and cells

**Fig 1:**
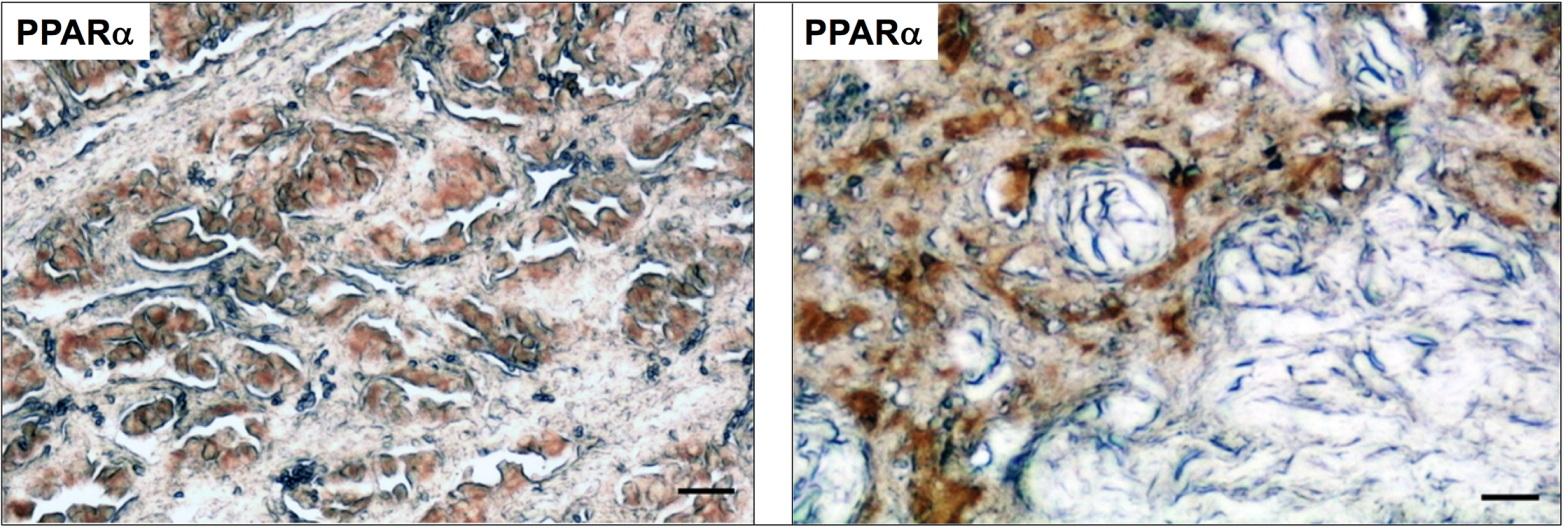
Immunohistochemical analysis of PPARα in HNPGL tissues. Tumor sections derived from two different HNPGL patients show positive immunostaining for PPARα in paraganglioma cell clusters, while the supporting stroma is negative. Bar = 80 μm.

**Fig 2:**
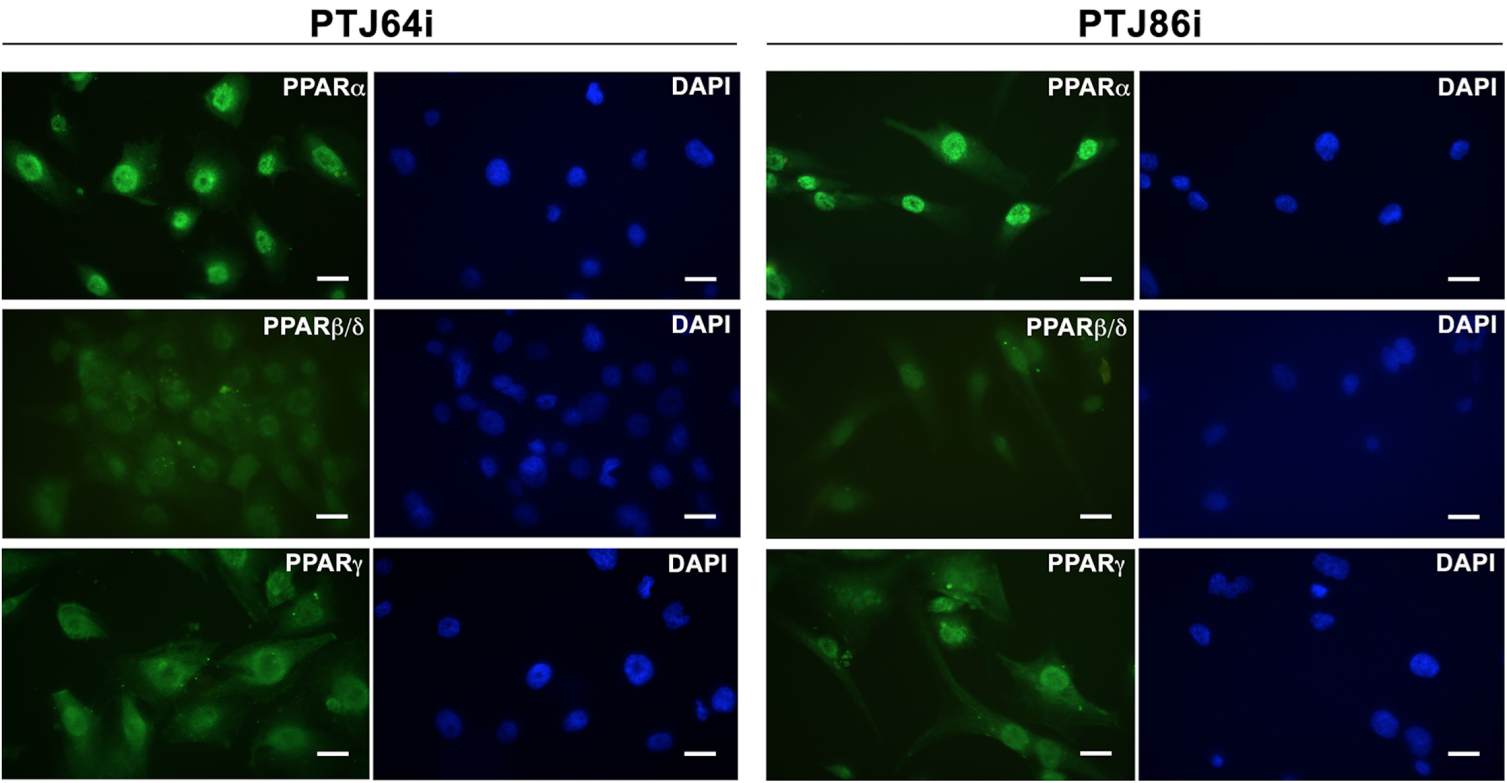
Immunofluorescence analysis of PPARs in HNPGL cells. PTJ64i (*left panels*) and PTJ86i (*right panels*) show high levels of PPARα nuclear expression. The fluorescence intensity for PPARα is stronger as compared to that for PPARβ/δ or PPARγ. Bar = 20 μm.

To evaluate whether PPARα is a relevant target in HNPGL we analyzed by immunohistochemistry whether this receptor is expressed in HNPGL patient tissues. As shown in Fig 1 PPARα appears to be expressed in HNPGLs as indicated by the positive staining for this receptor in formalin-fixed, paraffin-embedded tumor sections from two different HNPGL patients. We also evaluated by immunofluorescence the PPARs expression in HNPGL cells. Both PTJ64i and PTJ86i HNPGL cell lines showed an intense nuclear staining for PPARα, indicating that this protein is highly expressed in HNPGL cells (Fig 2). The fluorescence intensity for PPARα was higher as compared to that for PPARβ/δ and γ isoforms.

### Effects of PPARα agonist or antagonist on viability of HNPGL cells

**Fig 3:**
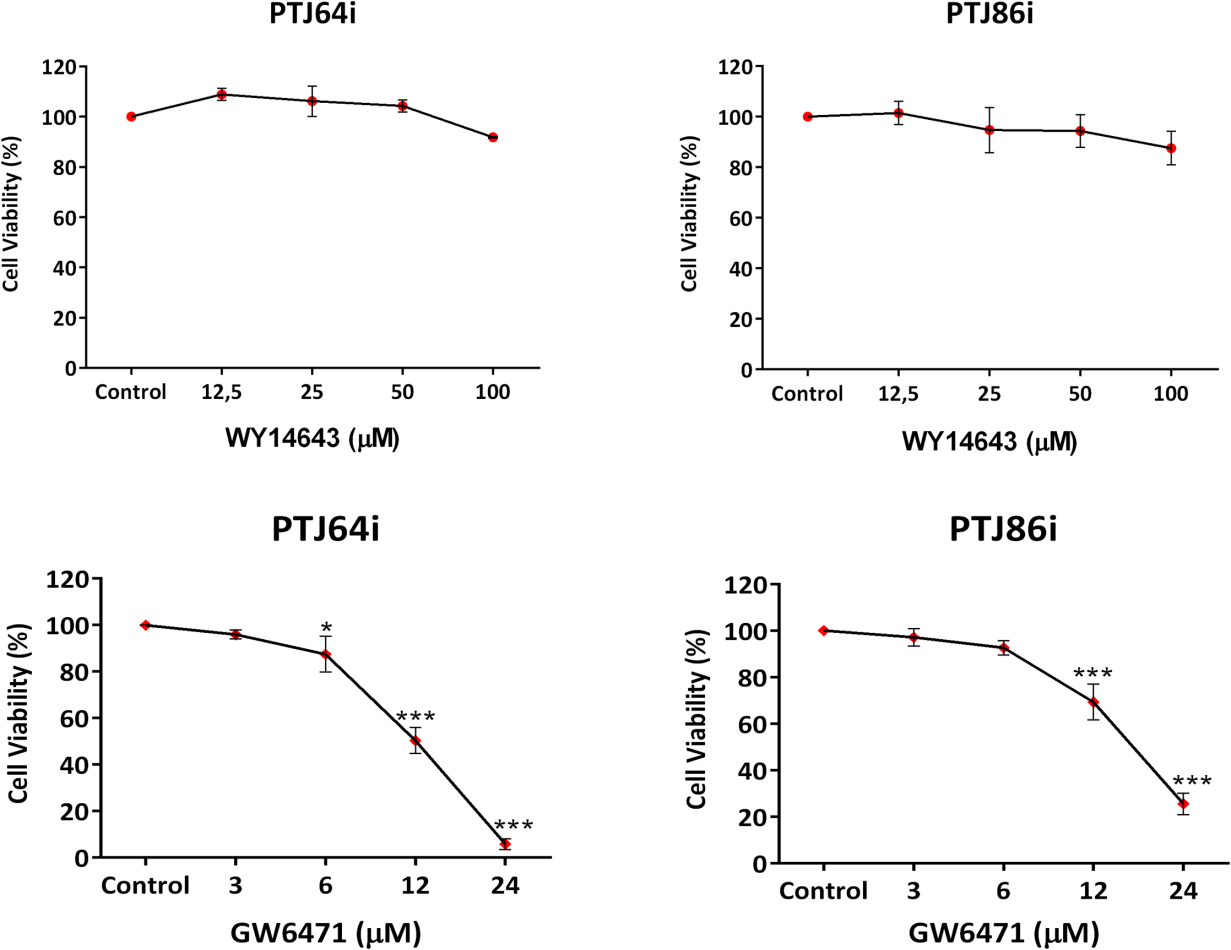
Effect of the PPARα agonist WY14643, or antagonist GW6471 on cell viability in PTJ64i and PTJ86i cell lines. Cells were incubated for 72 hours with WY14643 or GW6471 at the indicated concentrations, or with 0.16% DMSO vehicle (*control*). GW6471 significantly inhibited viability in both cell lines (IC_50_ of 10 μM in PTJ64i and 16 μM in PTJ86i). Data shown are the means ±SD of three experiments with quintuplicate determinations. *Significant differences between control and each drug concentration (*p<0.05; **p<0.01; ***p<0.001).

Based on the observation that PPARα was highly expressed in HNPGL cells we tested whether treatments with a specific PPARα agonist or antagonist would affect cell viability. Incubation of both PTJ64i and PTJ86i cell lines for 72 hours with the specific PPARα agonist WY14643, at concentrations ranging from 12.5 to 100 μM, did not affect cell viability as assessed by MTT assay (Fig 3 and S1 Table). Conversely, incubation with the specific PPARα antagonist GW6471 for 72 hours, at concentrations ranging from 3 to 24 μM, significantly reduced cell viability in a dose-dependent fashion in both PTJ64i and in PTJ86i cell lines (Fig 3 and S1 Table).

**Fig 4:**
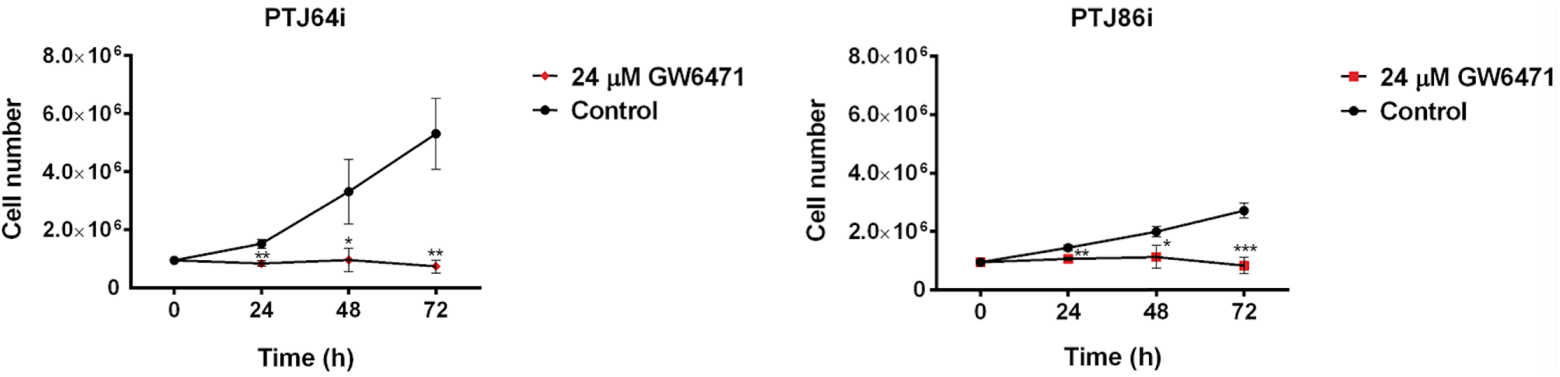
Growth curves of PTJ64i and PTJ86i cells treated with GW6471. Cell number was measured over a 72-hour time course treatment with 24 μM GW6471 or with vehicle control. Data shown are the means ±SD of three to five determinations (*p<0.05; **p<0.01; ***p<0.001).

Considering the effect of the antagonist on cell viability, we tested the impact of 24 μM GW6417 on PTJ64i and PTJ86i cell growth. Growth rate in the absence of treatment with GW6471 appeared to be faster for PTJ64i as compared to PTJ86i as reflected by cell counts at 24, 48 and 72 hours (Fig 4 and S2 Table). Despite these differences in the intrinsic growth rates, treatment with GW6471 markedly and significantly inhibited cell growth in both cell cultures at 24, 48 and 72 hours in a similar fashion, as compared to vehicle (Fig 4 and S2 Table).

### GW6471 affects cell cycle in HNPGL cells

**Fig 5:**
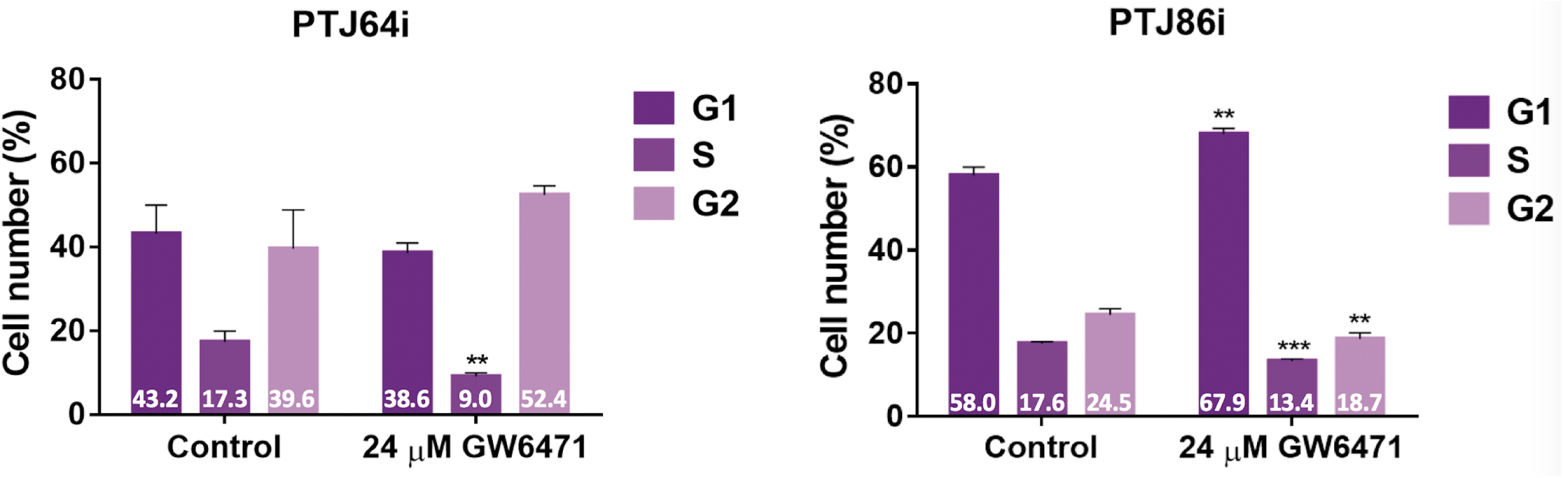
Effect of GW6471 on PTJ64i and PTJ86i cell cycles. The histograms show the mean percentage of cells (values inside the bars) in the different cell phases after a 24-hour treatment with 24 μM GW6471 as compared to control (**p<0.01; ***p<0.001).

To investigate whether the decreased cell viability observed after GW6471 treatment could be due to decreased proliferation we evaluated by flow cytometry whether the PPARα antagonist altered the proportions of cells in different cell cycle phases. The analysis was conducted after incubation of both cell lines with vehicle or 24 μM GW6471 for 24 hours. After a 24-hour treatment with GW6471, the percentage of PTJ64i cells in S phase was significantly reduced as compared to untreated cells (9% vs. 17.27% respectively; *p*<0.01) and the percentage of cells in the G2/M phase tended to increase (from 39.56% to 52.41%) (Fig 5 and S3 Table). This observation shows that the decreased cell viability observed by MTT in PTJ64i cells after treatment with GW6471 is associated with cell cycle inhibition. After a 24-hour treatment with GW6471, the percentage of PTJ86i cells in G0/G1 phase was significantly increased as compared to untreated cells (67.9% vs. 57.96% respectively; *p*<0.01). Conversely, after treatment a decrease in the percentage of cells in S phase (from 17.59% in untreated to 13.38% in treated; *p*<0.001) and in G2/M phase (from 24.45% to 18.71%; *p*<0.01) was observed (Fig 5 and S3 Table). Therefore, also in PTJ86i the decreased viability observed by MTT after GW6471 treatment is related to cell cycle inhibition, although the effects shown in this cell line appeared distinct from those observed in PTJ64i.

**Fig 6:**
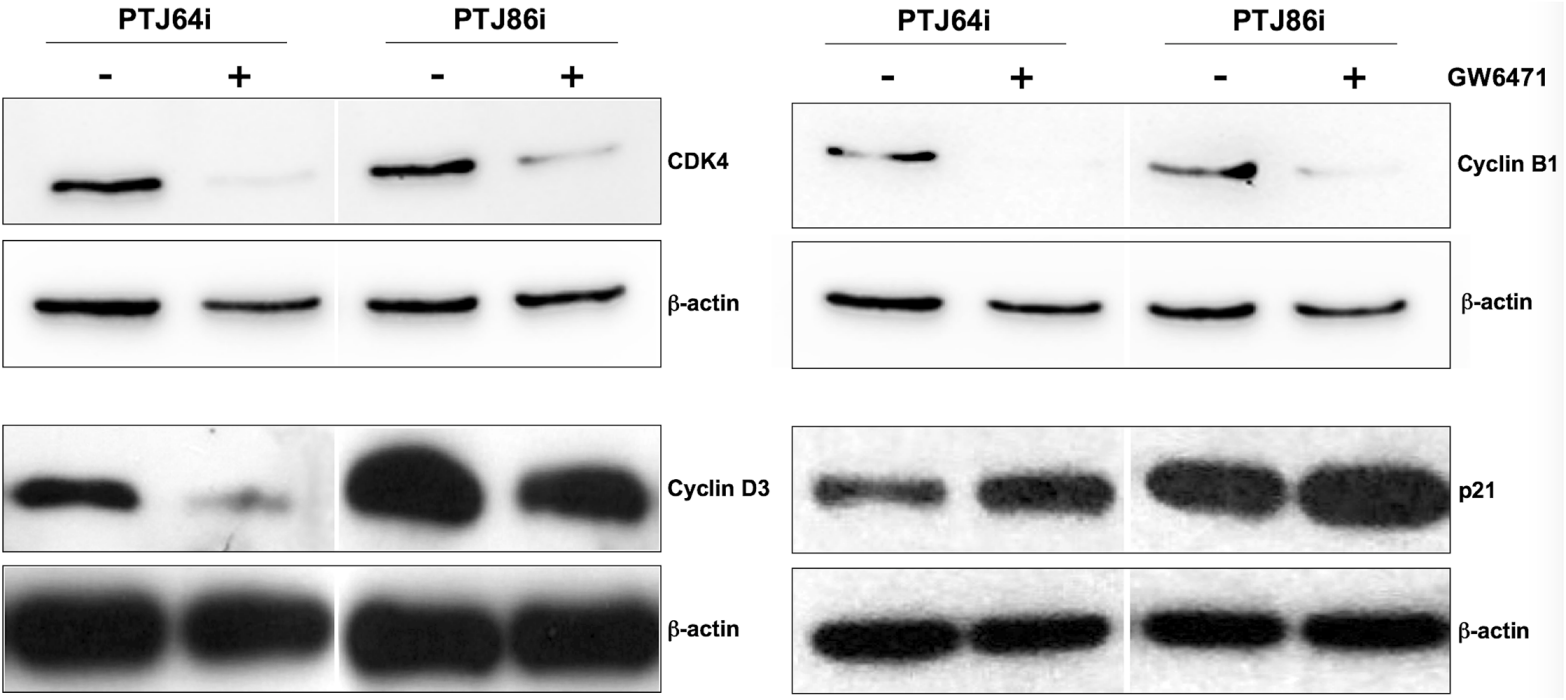
Effect of GW6471 on the expression of relevant cell cycle proteins in PTJ64i and PTJ86i cell lines. Expression of relevant cell cycle proteins after incubation of cells for 24 hours with 24 μM GW6471 was analyzed by Western blot using antibodies directed against the indicated proteins.

To analyze the mechanism of cell cycle arrest induction by GW6471, we measured the levels of CDK4, cyclin D3, cyclin B1 and p21 cell cycle regulators. Both cell lines were treated for 24 hours with 24 μM GW6471 and then the cell lysates were immunoblotted with the appropriate antibodies. In agreement with the cell cycle perturbation observed by flow cytometry, after GW6471 treatment CDK4, cyclin D3 and cyclin B1 expression was markedly decreased, whereas the expression of the universal cell cycle inhibitor p21 was increased in both cell lines (Fig 6).

### GW6471 promotes apoptosis in HNPGL cells

**Fig 7:**
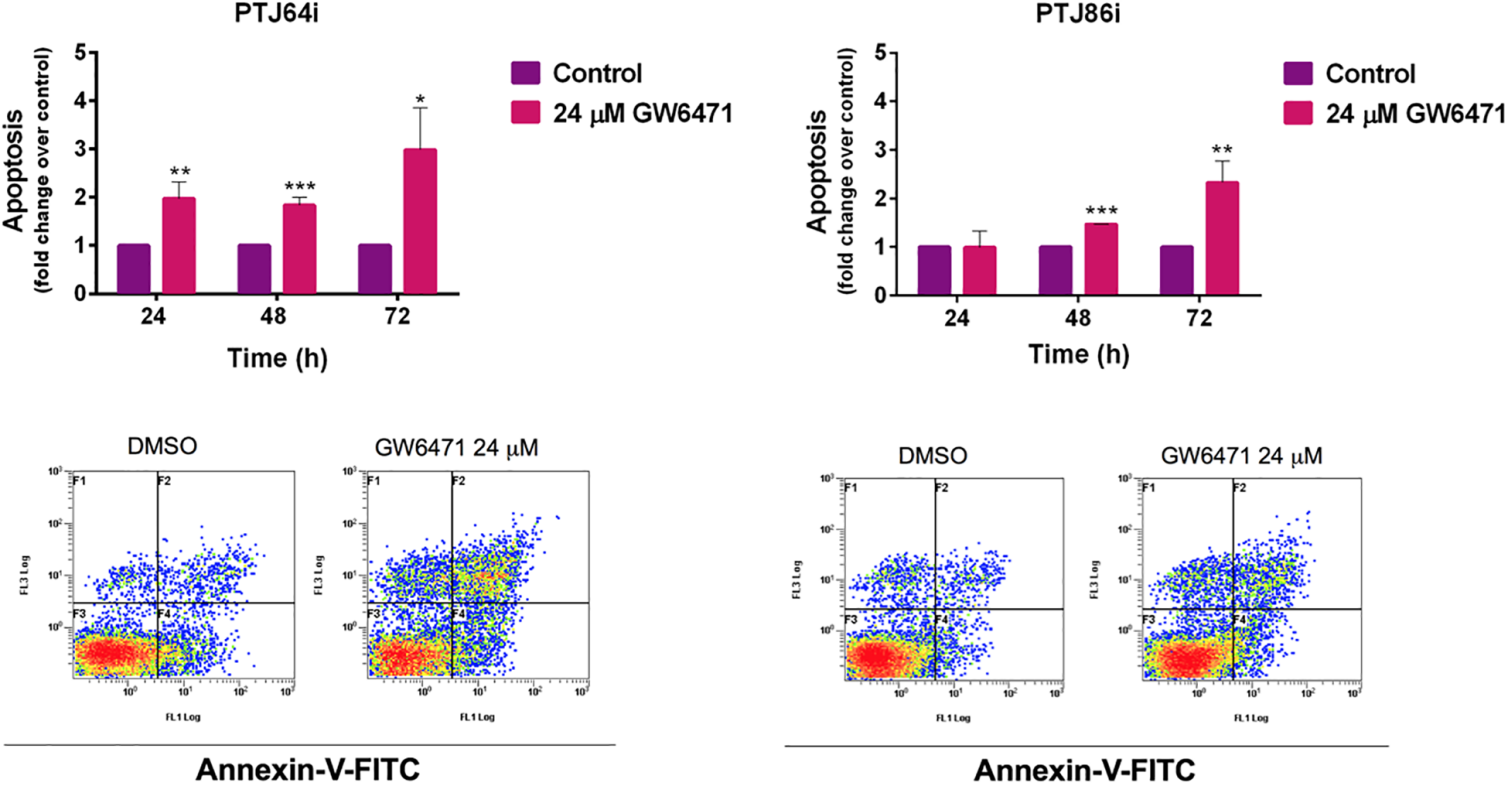
Apoptosis in PTJ64i and PTJ86i cells treated with 24 μM GW6471 for 24, 48 or 72 hours. Values represented in the histograms (*top*) are the means ±SD of at least two independent determinations (*p<0.05; **p<0.01; ***p<0.001). Dot plots (*bottom*) show representative experiments after a 72-hour treatment with 24 μM GW6471.

**Fig 8:**
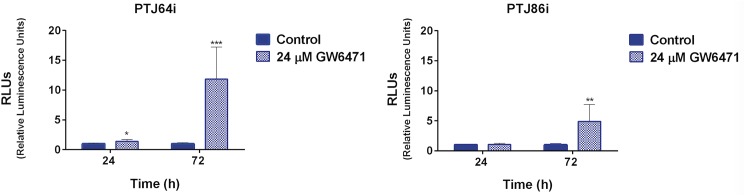
Caspase 3/7 activity in PTJ64i and PTJ86i cells treated with 24 μM GW6471 for 24 and 72 hours. Values represented in the histograms are the means ±SD of six determinations (*p<0.05; **p<0.01; ***p<0.001).

To analyze whether, in addition to cell cycle inhibition, the decreased cell viability observed after GW6471 treatment was due in part to apoptosis we evaluated Annexin-V staining by flow cytometry after treatment for 24, 48 and 72 hours. GW6471 treatment resulted in a significant induction of apoptosis in PTJ64i, with a sharper increment of apoptotic cells at 72 hours as compared to 24 and 48 hours (Fig 7 and S4 Table). A significant, but less pronounced, increment of apoptotic cells was observed also with PTJ86i after a 72h treatment with GW6471. Caspase 3/7 analysis confirmed the results obtained by flow cytometry, showing a significant increase of caspase 3/7 activity levels as compared to vehicle control (Fig 8 and S5 Table). These results indicate that programmed cell death contributes to the reduced viability induced by GW6471 in HNPGL cells.

### GW6471 represses the PI3K/GSK3/β-catenin pathway in HNPGL cell lines

**Fig 9:**
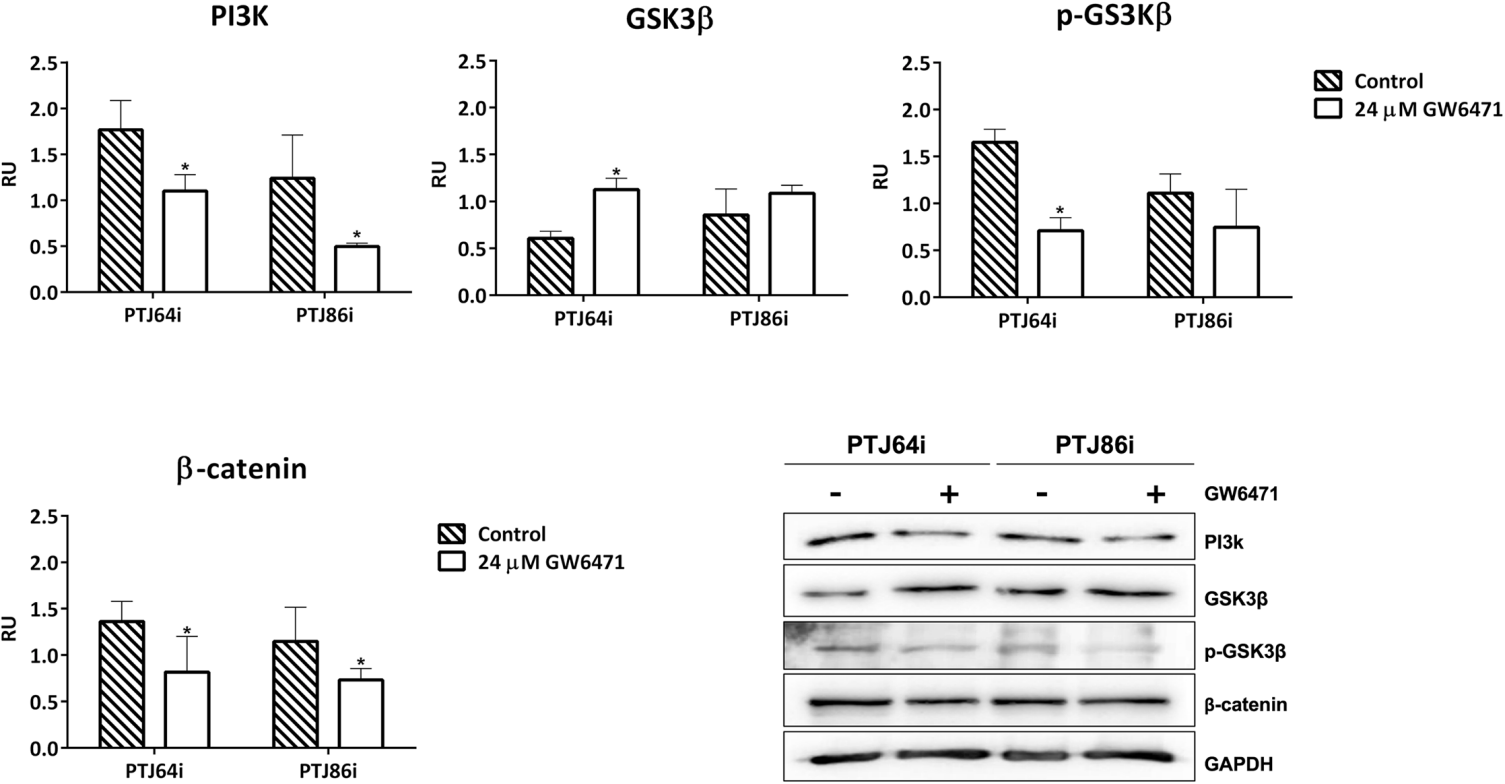
Effect of GW6471 on PI3K/GSK-3β/β-catenin signaling pathway in PTJ64i and PTJ86i cell lines. Histograms of normalized densitometric analyses and representative western blotting for PI3K, GSK3β, p-GSK3β and β-catenin in HNPGL cells treated with 24 μM GW6471 for 72 hours are shown. The relative densities of the immunoreactive bands were determined and normalized with respect to GAPDH (loading control) using a semiquantitative densitometric analysis (Kodak ID Image Analysis Software, Rochester, NY, USA). Values are expressed as relative units (RU). Each bar represents the mean ± SD of two up to five independent determinations (*p<0.05).

Western Blot analysis revealed that GW6471 treatment decreased phosphoinositide 3-kinase (PI3K) protein expression in both HNPGL cell lines. In addition, GW6471 treatment was associated with a trend of increased glycogen synthase kinase 3β (GSK3β) protein expression, as well decreased phosphorylation of GSK3β at Ser9 (the inactivated form of GSK3β) (Fig 9 and S6 Table). Consistently, GW6471 decreased the expression of β-catenin in both PTJ64i and PTJ86i cells (Fig 9 and S6 Table). Collectively, these results indicate that PI3K/GSK3/β-catenin signal was repressed by GW6471 in HNPGL.

### GW6471 inhibits clonogenicity and migration of HNPGL cells

**Fig 10:**
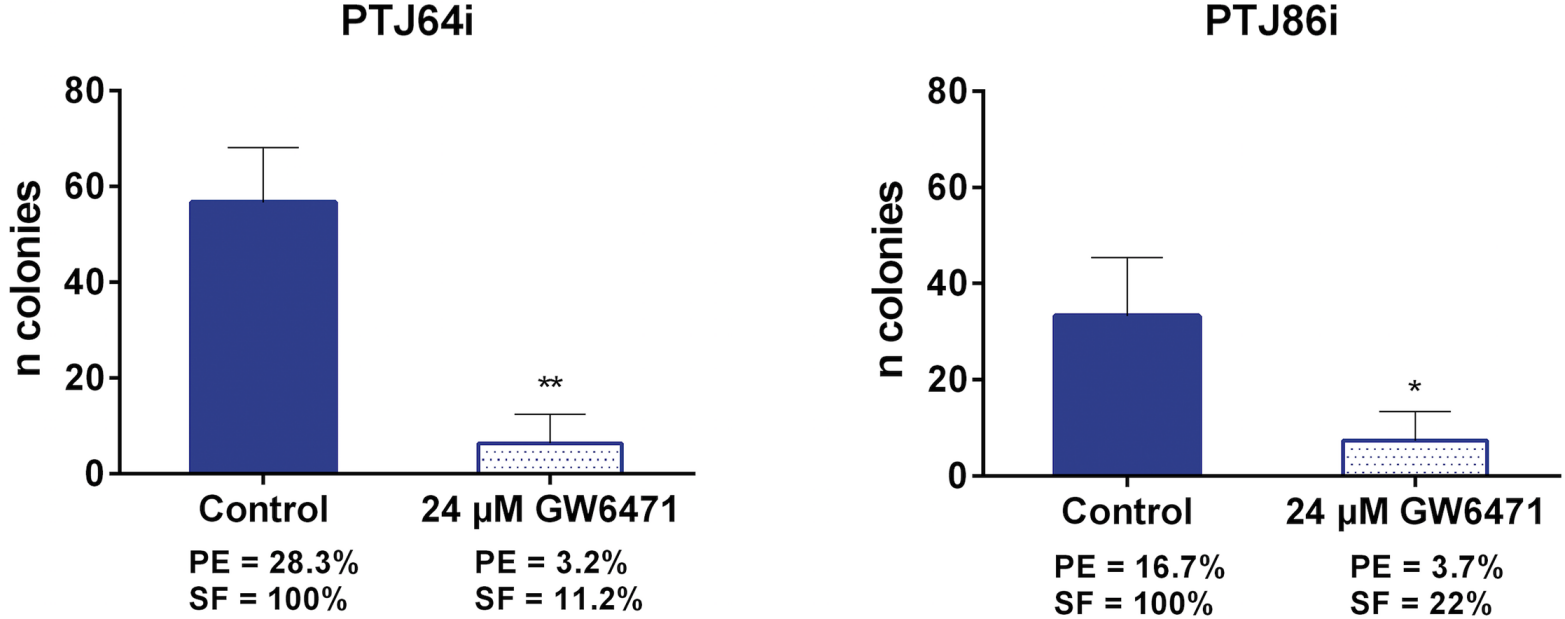
Effect of GW6471 on clonogenic activity of PTJ64i and PTJ86i cell lines. Data shown in the histograms are the means ±SD of three independent experiments (*p<0.05; **p<0.01). PE: plating efficiency [(# of colonies formed/# of cells plated)*100]; SF: surviving fraction [# of colonies formed *100/(# of cells plated *PE of control vehicle)].

**Fig 11:**
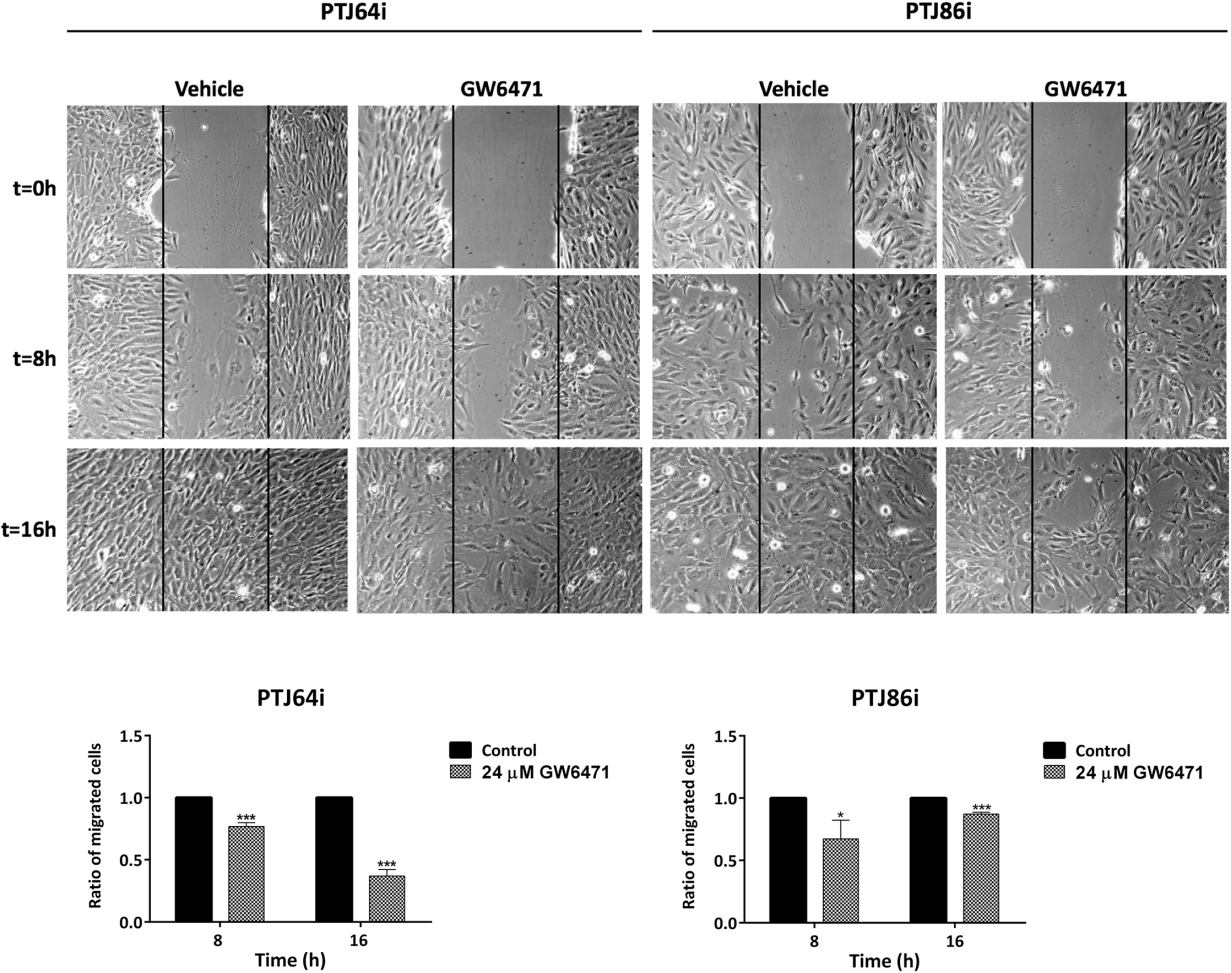
Wound healing assay. The effect of 24 μM GW6471 on HNPGL cell migration was evaluated by a monolayer wound-healing assay. Pictures of PTj64i and PTJ86i cells treated with vehicle or with GW6471 were taken at 0, 8 and 16 hours to analyze the dynamics of wound closure (vertical lines indicate wound edges). Histograms represent quantitative analyses of cell migration and are expressed as the ratio of the number of migrated cells in three fields after treatment as compared with vehicle (*p<0.05; ***p<0.001).

In the absence of treatment PTJ64i showed a higher plating efficiency than PTJ86i. Treatment with 24 μM GW6471 drastically impaired clonogenic activity of both cell cultures, as indicated by the reduced plating efficiency and surviving fraction as compared to vehicle control (Fig 10 and S7 Table). Overall, wound healing assay showed that 24 μM GW6471 had a modest effect on the migration of HNPGL cell lines at 8 hours (Fig 11 and S8 Table). This effect appeared more pronounced at 16 hours in PTJ64i.

## Discussion

HNPGLs are rare neoplasms of the paraganglia that cause important morbidity. At present, surgery is the only effective therapeutic option. Thus, novel therapeutic targets and molecules that could be exploited in HNPGL treatment are highly needed.

Targeting aberrant cell growth and metabolic pathways for cancer treatment is currently of great interest. In this regard, PPARs are ligand-activated transcription factors that are often deregulated in tumors [8]. In particular, the PPARα isoform appears to play an important role in the biology of different tumors where it may act as a tumor suppressor or an oncoprotein depending on cancer type. Tumor suppression by PPARα agonists has been reported in some cancers [27, 28], while PPARα overexpression has been found to lead to progression in other cancers [9–12, 14, 29]. Considering that PPARα is a candidate therapeutic target in some tumors and that its role in HNPGLs was never studied before we analyzed the expression of this nuclear receptor in HNPGL and tested the effects of a specific PPARα agonist (WY14643) or antagonist (GW6471).

We observed an intense immunoreactivity for PPARα in HNPGL tumors, suggesting that this receptor has an important role also in HNPGL. A pronounced nuclear expression of PPARα was confirmed in both PTJ64i and PTJ86i HNPGL cell lines. Notably, the fluorescence intensity for PPARα in HNPGL cell lines was comparable to what we have previously observed in glioblastoma [12], a tumor overexpressing PPARα whose viability is also affected by a PPARα antagonist [20]. The overexpression of PPARα and its nuclear localization suggested that antagonizing this protein might have antitumor effects in HNPGL. In line with this possibility the agonist WY14643 did not affect cell viability, whereas GW6471 reduced viability in both PTJ64i and PTJ86i in a dose-dependent way. GW6471 also reduced growth of both HNPGL cell cultures. These findings obtained with GW6471 in HNPGL cells are in line with those previously reported for kidney cancer cells, where the same molecule reduced cell viability by specific inhibition of PPARα [14]. In that study, the effects of GW6471 on the growth and viability of kidney cancer cells were related to interference with cell cycle and induction of apoptosis. Also in our study, the reduced viability observed in both HNPGL cell lines after GW6471 treatment was related to an interference with cell cycle progression and apoptosis. In this regard, GW6471 altered the expression of cell cycle proteins in a similar fashion in both HNPGL cell lines and affected cell cycle progression. Nevertheless, the pattern of cell cycle inhibition observed in PTJ64i by flow cytometry appeared to be distinct from that observed in PTJ86i. This might reflect the intrinsic growth rate of the two cell lines, which appeared to be lower in PTJ86i. Regarding the effects of GW6471 on apoptosis this drug induced caspase-dependent apoptosis in both PTJ64i and PTJ86i cell lines, with a significant and more evident effect on apoptosis after a 72-hour treatment. Also the clonogenic ability of HNPGL cells, which constitutes a direct measure of cell self-renewal capacity [25], was markedly reduced after GW6471 treatment, indicating that the drug had a profound impact on this capacity. Conversely, the effect of GW6471 on HNPGL cell migration was less marked.

The β-catenin transcriptional program pathway plays a crucial role in several processes relevant to tumor biology [30]. The level of intracellular β-catenin is regulated by PI3K via GSK3β, which phosphorylates β-catenin to promote its ubiquitin-proteasome degradation [31]. Our data show that GW6471 treatment was associated with decreased expression of PI3K. We also observed the reduction of β-catenin protein expression in HNPGL cells, which was consistent with the alterations of GSK3β expression and phosphorylation observed in response to GW6471. Taken together, our data show that the inhibitory effect of GW6471 on HNPGL cell viability was due, at least in part, to the inhibition of the PI3K/GSK3β/β-catenin signaling pathway.

Overall, our results indicate a remarkable antitumor effect of GW6471 on HNPGL *in vitro*. Future studies will be necessary to test whether this molecule is active also in animal models of HNPGL. In this regard, it is worth noting that PPARα inhibition by GW6471 was recently reported to attenuate kidney cancer growth in a xenograft mouse model, with no adverse effects in the animals [32].

In conclusion, we show that PPARα is overexpressed in HNPGL and that the PPARα antagonist GW6471 is able to reduce viability in this unique model of HNPGL cells by interfering with cell cycle and by inducing apoptosis. The mechanisms affecting HNPGL cell viability involve repression of the PI3K/GSK3β/β-catenin pathway. Therefore, PPARα could represent a novel therapeutic target for this rare chemoresistant tumor. To our knowledge this is the first study analyzing the effects of molecules with therapeutic potential in an *in vitro* model of HNPGL cells.

## Supporting information

S1 Table Effect of WY14643 (A) or GW6471 (B) treatments on cell viability (%) as assessed by MTT.
(XLSX)

S2 Table Effect of GW6471 treatment on cell growth. (XLSX)

S3 Table Effect of GW6471 treatment on cell cycle as assessed by flow cytometry. (XLSX)

S4 Table Effect of GW6471 treatment on apoptosis as assessed by flow cytometry. (XLSX)

S5 Table Effect of GW6471 treatment on caspase 3/7 activity. (XLSX)

S6 Table Effect of GW6471 treatment on PI3K/GSK3β/β-catenin pathway. (XLSX)

S7 Table Effect of GW6471 treatment on colony formation. (XLSX)

S8 Table Effect of GW6471 treatment on wound healing. (XLSX)
